# Combined prognostic effect of PD-L1 expression and immunoscore in microsatellite-unstable advanced gastric cancers

**DOI:** 10.18632/oncotarget.19439

**Published:** 2017-07-22

**Authors:** Kyung-Ju Kim, Han Kwang Yang, Woo Ho Kim, Gyeong Hoon Kang

**Affiliations:** ^1^ Laboratory of Epigenetics, Cancer Research Institute, Seoul National University College of Medicine, Seoul 03080, Republic of Korea; ^2^ Department of Pathology, Yeungnam University College of Medicine, Daegu 42415, Republic of Korea; ^3^ Department of General Surgery, Seoul National University College of Medicine, Seoul 03080, Republic of Korea; ^4^ Department of Pathology, Seoul National University College of Medicine, Seoul 03080, Republic of Korea

**Keywords:** gastric cancer, microsatellite instability, PD-L1, immunoscore, prognosis

## Abstract

**Background:**

The aim of this study was to evaluate how programmed death-ligand-1 (PD-L1) expression is linked to the immunoscore in the context of the tumor microenvironment and to assess the differential prognostic value of PD-L1 expression according to the immunoscore in 153 patients with microsatellite instability-high (MSI-H) advanced gastric cancer (GC).

**Results:**

We found that T-PD-L1 (+) and I-PD-L1 (+) were significantly associated with a high immunoscore. The integrated PD-L1 expression of tumor and immune cells was not significantly correlated with the overall survival (OS) of patients. However, a combined survival analysis of PD-L1 expression and immunoscore revealed four distinct subgroups with a statistically significant difference in OS. That is, the PD-L1 (+)/immunoscoreLow group showed the worst and the PD-L1 (+)/immunoscoreHigh group showed the best prognosis. Furthermore, a multivariate analysis revealed that the combined status of PD-L1 expression and immunoscore was an independent and significant prognostic factor for OS in patients with MSI-H GC.

**Materials and Methods:**

The immunoscore was quantified by the number of high-density areas of CD3+ and CD8+ tumor infiltrating lymphocytes both in the tumor regions and compartments (i.e., epithelial and stromal compartments of the tumor center and the invasive front), the scores of which range from I0 to I8. By using immunohistochemistry, the expression of PD-L1 was also analyzed in tumor cells (T-PD-L1) and immune cells (I-PD-L1) using four different cut-off values (1%, 5%, 10% and 50%).

**Conclusions:**

Our study revealed that PD-L1 expression is associated with the corresponding immunoscore and that the immunoscore can be a relevant marker for the determination of the prognostic role of PD-L1 expression in MSI-H GCs.

## INTRODUCTION

Gastric cancer (GC) is ranked as the fifth most common malignancy in the world and is the third most common cause of cancer-related death [1]. GC is a heterogeneous disease in terms of molecular carcinogenesis, and microsatellite instability (MSI) accounts for approximately 10% of GCs [2, 3]. MSI refers to genome-wide alterations in the number of repeated nucleotides known as microsatellites, which may be located in both coding and non-coding regions of genes; these alterations result in frameshift mutations. MSI-high (MSI-H) GCs are generally characterized by some distinct clinicopathological features, including an increased number of tumor infiltrating lymphocytes (TILs), compared with microsatellite-stable (MSS) GCs [4].

Programmed cell death-ligand 1 (PD-L1) is a 40-kDa type 1 transmembrane protein that is involved in the immunoregulatory system during certain conditions such as autoimmune disease, pregnancy, allograft rejection, and cancer [5]. Activation of the programmed cell death-1 (PD-1)/PD-L1 signaling pathway leads to an immunosuppressive tumor microenvironment, which results in immune evasion by tumor cells [6]. Thus, inhibition of the PD-1/PD-L1 signaling axis may be a candidate strategy in cancer immunotherapy. Many clinical trials have revealed that anti-PD-1/PD-L1 therapy is effective against various types of tumors, including malignant melanoma, non-small cell lung cancer, and renal cell carcinoma [7, 8]. A phase Ib clinical trial showed that pembrolizumab, an anti-PD-1 antibody, displays promising antitumor activity against GC and has manageable toxicities [9]. A recent phase II trial reported that mismatch-repair status predicts a survival benefit during blockade of the immune checkpoint system in colorectal cancer (CRC) patients [10]. In this regard, several studies of CRC and GC demonstrated that PD-L1 expression in tumor cells and infiltrating immune cells is significantly associated with the MSI-H phenotype and a high density of tumor-associated immune cells [11–14].

Many studies support the concept that TILs have a prognostic value and the “immunoscore” has been demonstrated to be a powerful prognostic indicator. Evidence also indicates that these might be equivalent to the American Joint Committee on Cancer (AJCC)/ Union for International Cancer Control-tumor node metastasis (TNM) staging system in terms of their ability to predict the clinical outcome of patients with malignant tumors [15–17]. Previous studies revealed that a high immunoscore is associated with a longer disease-free survival and overall survival (OS) in several cancer types, especially in CRCs [18]. MSI-H cancers are considered to be highly immunogenic due to the accumulation of neo-antigens that are produced by a frameshift mutation in mismatch-repair-deficient conditions [11]. Therefore, MSI-H GCs are thought to provide an adequate platform for the evaluation of the relevance of tumor infiltrating immune cells and PD-L1 expression.

In the current study, we analyzed PD-L1 expression in the context of the tumor microenvironment and assessed 1) how PD-L1 expression is linked to the immunoscore and 2) the differential prognostic value of PD-L1 expression according to the immunoscore.

## RESULTS

### The prognostic implications of immunoscore in MSI-H GCs

**Figure 1:**
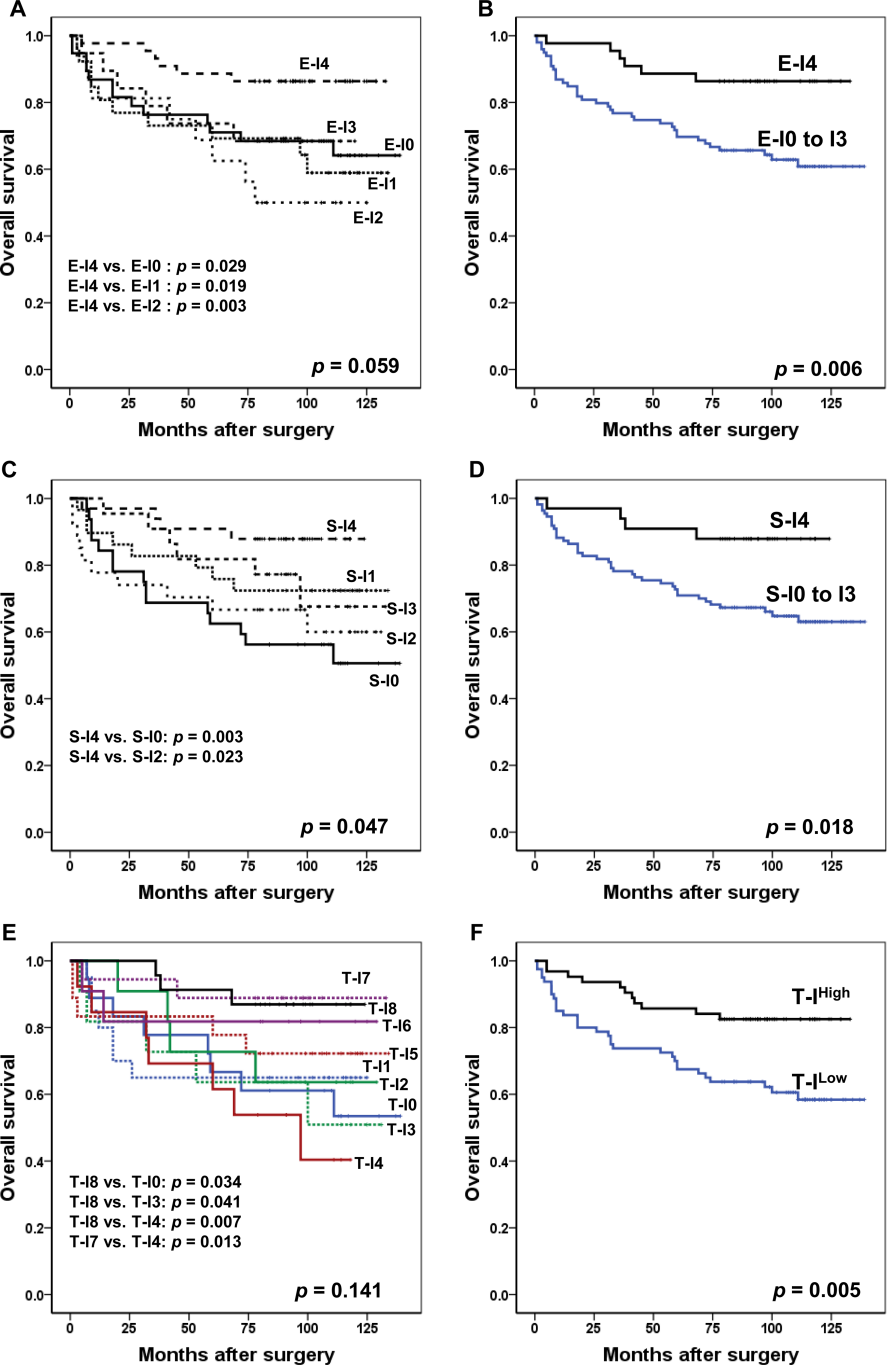
Kaplan-Meier survival analysis with log-rank test of the immunoscore (**A**) Survival curves for OS according to the E-I (No. of patients; E-I0, 38; E-I1, 26; E-I2, 16; E-I3, 19; E-I4, 44). (**B**) Survival curves for OS in patients with E-I0 to E-I3 (*n* = 99) vs. E-I4 (*n* = 44). (**C**) Survival curves for OS according to the S-I (No. of patients; S-I0, 32; S-I1, 29; S-I2, 27; S-I3, 22; S-I4, 33). (**D**) Survival curves for OS in patients with S-I0 to S-I3 (*n* = 110) vs. S-I4 (*n* = 33). (**E**) Survival curves for OS according to the T-I (No. of patients; T-I0, 18; T-I1, 20; T-I2, 18; T-I3, 11; T-I4, 13; T-I5, 11; T-I6, 11; T-I7, 18; T-I8, 23). (**F**) Survival curves for OS in patients with T-I^High^ (*n* = 63) vs. T-I^Low^ (*n* = 80). Abbreviations: OS, overall survival; E-I, immunoscore in epithelial compartment; S-I, immunoscore in stromal compartment; T-I, total immunoscore.

In Kaplan-Meier analysis, repartitioning of cases according to the E-I resulted in borderline significance for discriminating the clinical outcome among five subgroups (*p* = 0.059). Although the patients with E-I4 showed the best prognosis, higher scores did not guarantee a better prognosis among patients with E-I0, E-I1, E-I2, and E-I3 (Figure 1A). When we grouped the patients into two subgroups (E-I0 to E-I3 vs. E-I4), the E-I4 group had a significant survival advantage in OS (*p* = 0.006) (Figure 1B). Regarding S-I, the S-I4 and S-I0 groups had the best and the worst clinical outcomes, respectively (*p* = 0.047) (Figure 1C). However, the S-I1 group had the second-best prognosis. When the cases were categorized into two subgroups (S-I0 to S-I3 vs. S-I4), the S-I4 group had prolonged OS compared to that of the rest (*p* = 0.018) (Figure 1D). Although the mortality risk was not proportionally increased with a decrease in T-I (*p* = 0.141) (Figure 1E), tumors could be largely divided into two subgroups based on the T-I; T-I^Low^ (T-I0 to T- I4) or T-I^High^ (T-I5 to T-I8) (*p* = 0.005) (Figure 1F). In multivariate analysis with adjustments to lymphatic invasion, vascular invasion, perineural invasion, Ming classification, TNM stage, and T-I (which were significant factors in the univariate analysis, Supplementary Table 1), T-I remained an independent prognostic indicator (*p* = 0.044) (Table 1).

**Table 1:** Multivariate analysis of OS among patients with MSI-H GCs including T-I

Variable		Multivariate analysis	
	*n*	HR (95% CI)	*p*-value
Ming			
Expanding	37	1 (Reference)	0.070
Infiltrative	106	2.644 (0.924–7.560)	
Lymphatic invasion			
Absent	50	1 (Reference)	0.626
Present	93	1.233 (0.531–2.859)	
Vascular invasion			
Absent	120	1 (Reference)	0.060
Present	23	2.002 (0.972–4.123)	
Perineural invasion			
Absent	89	1 (Reference)	0.592
Present	54	1.189 (0.631–2.242)	
AJCC stage			
I/II	85	1 (Reference)	<0.001
III/IV	58	4.650 (2.178–9.928)	
T-I^a^			
T-I^Low^ (T-I0 to T-I4)	80	1 (Reference)	0.044
T-I^High^ (T-I5 to T-I8)	63	0.047 (0.227–0.978)	

^a^Included only for patients with available TMA data.

Abbreviations: OS, overall survival; MSI-H, microsatellite instability-high; GC, gastric cancer; HR, hazard ratio; CI, confidence interval; AJCC, American American Joint Committee on Cancer; T-I, total immunoscore; TMA, tissue macroarray.

### Clinicopathological features associated with PD-L1 expression in MSI-H GCs

**Figure 2:**
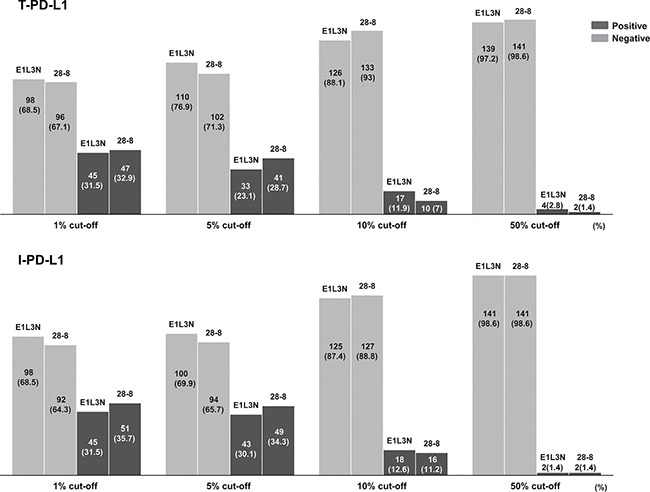
Frequencies of T-PD-L1 and I-PD-L1 expression status for two different monoclonal antibodies (E1L3N and 28-8)

The expression status of PD-L1 was evaluated using E1L3N in 143 MSI-H GC samples based on four different cut-off values (1%, 5%, 10%, and 50%). The frequency of the positive and negative expression of PD-L1 at each cut-off value is shown in Figure 2. For the 5% cut-off value, PD-L1 positivity in tumor cells (T-PD-L1 (+)) and immune cells (I-PD-L1 (+)) was detected in 33 (23.1%) and 43 (30.1%) of 143 MSI-H GCs, respectively. Double positivity in tumor cells and immune cells was observed in 18 cases (12.6%). The T-PD-L1 (+) phenotype was closely associated with a diffuse or mixed tumor type according to the Lauren classification (*p* = 0.033), less frequent lymphatic invasion (*p* = 0.002), lower TNM stage (*p* = 0.030), and a high immunoscore (*p* = 0.003) compared with T-PD-L1 (−) phenotype (Table 2). I-PD-L1 (+) tumors were significantly correlated with the expanding type of GC according to the Ming classification (*p* = 0.042), less frequent lymphatic invasion (*p* = 0.001), less frequent perineural invasion (*p* = 0.019), less frequent LN metastasis (*p* = 0.019), lower TNM stage (*p* = 0.006), and a high immunoscore (*p* < 0.001) (Table 2).

**Table 2:** Associations between PD-L1 expression and clinicopathological characteristics

Parameter	Case no.	T-PD-L1 (5% cut-off value)^a^			I-PD-L1 (5% cut-off value)^a^		
		Negative	Positive	*p*-value	Negative	Positive	*p*-value
Sex				0.119			0.316
Male	74	53 (48.2)	21 (63.6)		49 (49.0)	25 (58.1)	
Female	69	57 (51.8)	12 (36.4)		51 (51.0)	18 (41.9)	
Age (years)				0.620			0.927
≤ 60	44	35 (31.8)	9 (27.3)		31 (31.0)	13 (30.2)	
> 60	99	75 (68.2)	24 (72.7)		69 (69.0)	30 (69.8)	
Tumor differentiation				0.405			0.808
WD/MD	61	49 (44.5)	12 (36.4)		42 (42.0)	19 (44.2)	
PD/Other	82	61 (55.5)	21 (63.6)		58 (58.0)	24 (55.8)	
Ming				0.834			0.042
Expanding	37	28 (25.5)	9 (27.3)		21 (21.0)	16 (37.2)	
Infiltrative	106	82 (74.5)	24 (72.7)		79 (79.0)	27 (62.8)	
Lauren				0.033			0.304
Intestinal	76	65 (59.0)	11 (33.3)		55 (55.0)	21 (48.8)	
Diffuse	41	28 (25.5)	13 (39.4)		25 (25.0)	16 (37.2)	
Mixed	26	17 (15.5)	9 (27.3)		20 (20.0)	6 (14.0)	
Lymphatic invasion				0.002			0.001
Absent	50	31 (28.2)	19 (57.6)		26 (26.0)	24 (55.8)	
Present	93	79 (71.8)	14 (42.4)		74 (74.0)	19 (44.2)	
Vascular invasion				0.361			0.649
Absent	120	94 (85.5)	26 (78.8)		83 (83.0)	37 (86.0)	
Present	23	16 (14.5)	7 (21.2)		17 (17.0)	6 (14.0)	
Perineural invasion				0.550			0.019
Absent	89	67 (60.9)	22 (66.7)		56 (56.0)	33 (76.7)	
Present	54	43 (39.1)	11 (33.3)		44 (44.0)	10 (23.3)	
Tumor depth				0.653			0.072
T2/3	113	86 (78.2)	27 (81.8)		75 (75.0)	38 (88.4)	
T4	30	24 (21.8)	6 (18.2)		25 (25.0)	5 (11.6)	
LN metastasis				0.129			0.019
Absent	112	83 (75.5)	29 (87.9)		73 (73.0)	39 (90.7)	
Present	31	27 (24.5)	4 (12.1)		27 (27.0)	4 (9.3)	
AJCC stage				0.030			0.006
I/II	85	60 (54.5)	25 (75.8)		52 (52.0)	33 (76.7)	
III/IV	58	50 (45.5)	8 (24.2)		48 (48.0)	10 (23.3)	
T-I ^a^				0.003			< 0.001
T-I^Low^ (T-I0 to T-I4)	80	69 (62.7)	11 (33.3)		68 (68.0)	12 (27.9)	
T-I^High^ (T-I5 to T-I8)	63	41 (37.3)	22 (66.7)		32 (32.0)	31 (72.1)	

Values are presented as the number (%).

^a^Included only for patients with available TMA data.

Abbreviations: T-PD-L1, PD-L1 expression in tumor cells; I-PD-L1, PD-L1 expression in immune cells; WD, well differentiated; MD, moderately differentiated; PD, poorly differentiated; LN, lymph node; AJCC, American Joint Committee on Cancer; T-I, total immunoscore; TMA, tissue microarray.

### Prognostic significance of T-PD-L1 and I-PD-L1 expression status in MSI-H GCs

**Figure 3:**
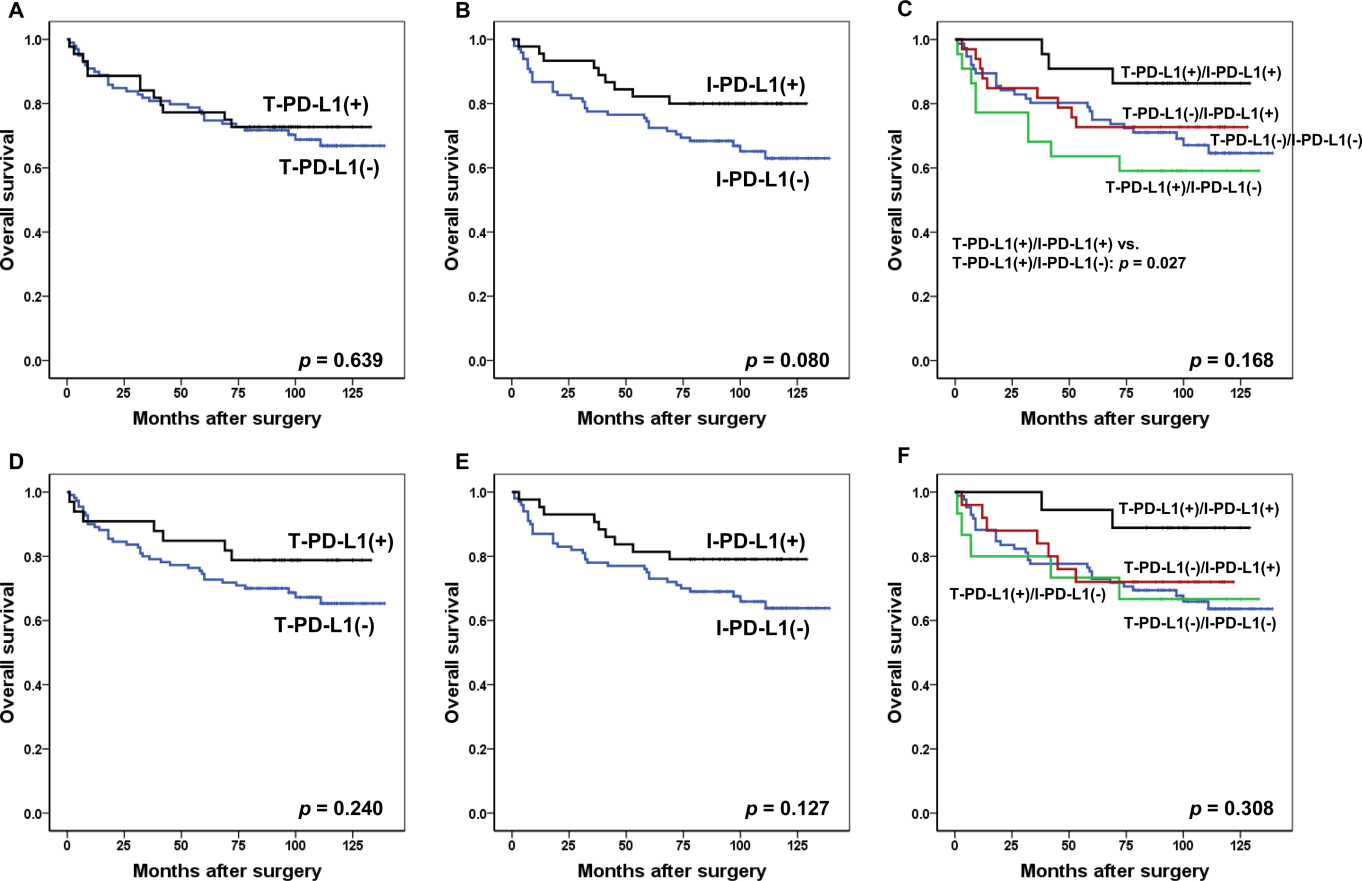
Kaplan-Meier survival analysis of T-PD-L1 and I-PD-L1 for 1% cut-off value (**A**–**C**) and 5% cut-off value (**D**–**F**). (A) Survival curves for OS according to PD-L1 expression in tumor cells (No. of patients; T-PD-L1 (+), 45; T-PD-L1 (−), 98). (B) Survival curves for OS according to PD-L1 expression in immune cells (No. of patients; I-PD-L1 (+), 45; I-PD-L1 (−), 98). (C) Survival curves for OS according to combined T-PD-L1 and I-PD-L1 expression status (No. of patients; T-PD-L1 (+)/I-PD-L1 (+), 23; T-PD-L1 (−)/I-PD-L1 (+), 22; T-PD-L1 (+)/I-PD-L1 (−), 22; T-PD-L1 (−)/I-PD-L1 (−), 76). (D) Survival curves for OS according to PD-L1 expression in tumor cells (No. of patients; T-PD-L1 (+), 33; T-PD-L1 (−), 110). (E) Survival curves for OS according to PD-L1 expression in immune cells (No. of patients; I-PD-L1 (+), 43; I-PD-L1 (−), 100). (F) Survival curves for OS according to combined T-PD-L1 and I-PD-L1 expression status (No. of patients; T-PD-L1 (+)/I-PD-L1 (+), 18; T-PD-L1 (−)/I-PD-L1 (+), 25; T-PD-L1 (+)/I-PD-L1 (−), 15; T-PD-L1 (−)/I-PD-L1 (−), 85). Abbreviations: T-PD-L1, PD-L1 expression in tumor cells; I-PD-L1, PD-L1 expression in immune cells; OS, overall survival.

Using 1%, 5%, 10%, and 50% cut-off values, Kaplan-Meier survival analysis was performed for 143 MSI-H GCs according to the expression status of PD-L1 using E1L3N. At any cut-off value, the difference in survival was not significant between patients with T-PD-L1 (+) and T-PD-L1 (−) or between I-PD-L1 (+) and I-PD-L1 (−) (Supplementary Table 2). To be more specific, at the 1% cut-off value, the difference in survival was not significant between patients with T-PD-L1 (+) and the T-PD-L1 (−) (*p* = 0.639) (Figure 3A). However, the I-PD-L1 (+) group showed a trend of advantage in survival over the I-PD-L1 (−) group (*p* = 0.080) (Figure 3B). In survival analysis for the combined prognostic effect of T-PD-L1 and I-PD-L1, patients with T-PD-L1 (+)/I-PD-L1 (+) and T-PD-L1 (+)/I-PD-L1 (−) had the best and worst clinical outcomes respectively, with borderline significance (*p* = 0.168) (Figure 3C). Notably, in subgroup analysis, the T-PD-L1 (+)/I-PD-L1 (+) group exhibited significantly better OS compared to the T-PD-L1 (+)/I-PD-L1 (−) group (*p* = 0.027). At 5% cut-off value, a similar trend for OS was observed. The expression status of T-PD-L1 and I-PD-L1 could not significantly discriminate the survival outcomes of these patients (*p* = 0.240 for T-PD-L1; *p* = 0.127 for I-PD-L1), although the tendency for a better survival outcome was observed in patients with T-PD-L1 (+) compared to those with T-PD-L1 (−) and in patients with I-PD-L1 (+) compared to those with I-PD-L1 (−) (Figure 3D and 3E). Furthermore, a combination of T-PD-L1 and I-PD-L1 failed to demonstrate a survival difference among the four subgroups (T-PD-L1 (+)/I-PD-L1 (+) vs. T-PD-L1 (+)/I-PD-L1 (−) vs. T-PD-L1 (−)/I-PD-L1 (+) vs. T-PD-L1 (−)/I-PD-L1 (−)) (*p* = 0.308), despite the observation that patients in the T-PD-L1 (+)/I-PD-L1 (+) groups experienced the longest OS compared with the other groups (Figure 3F).

### Prognostic value of PD-L1 expression combined with the immunoscore in MSI-H GCs

**Figure 4:**
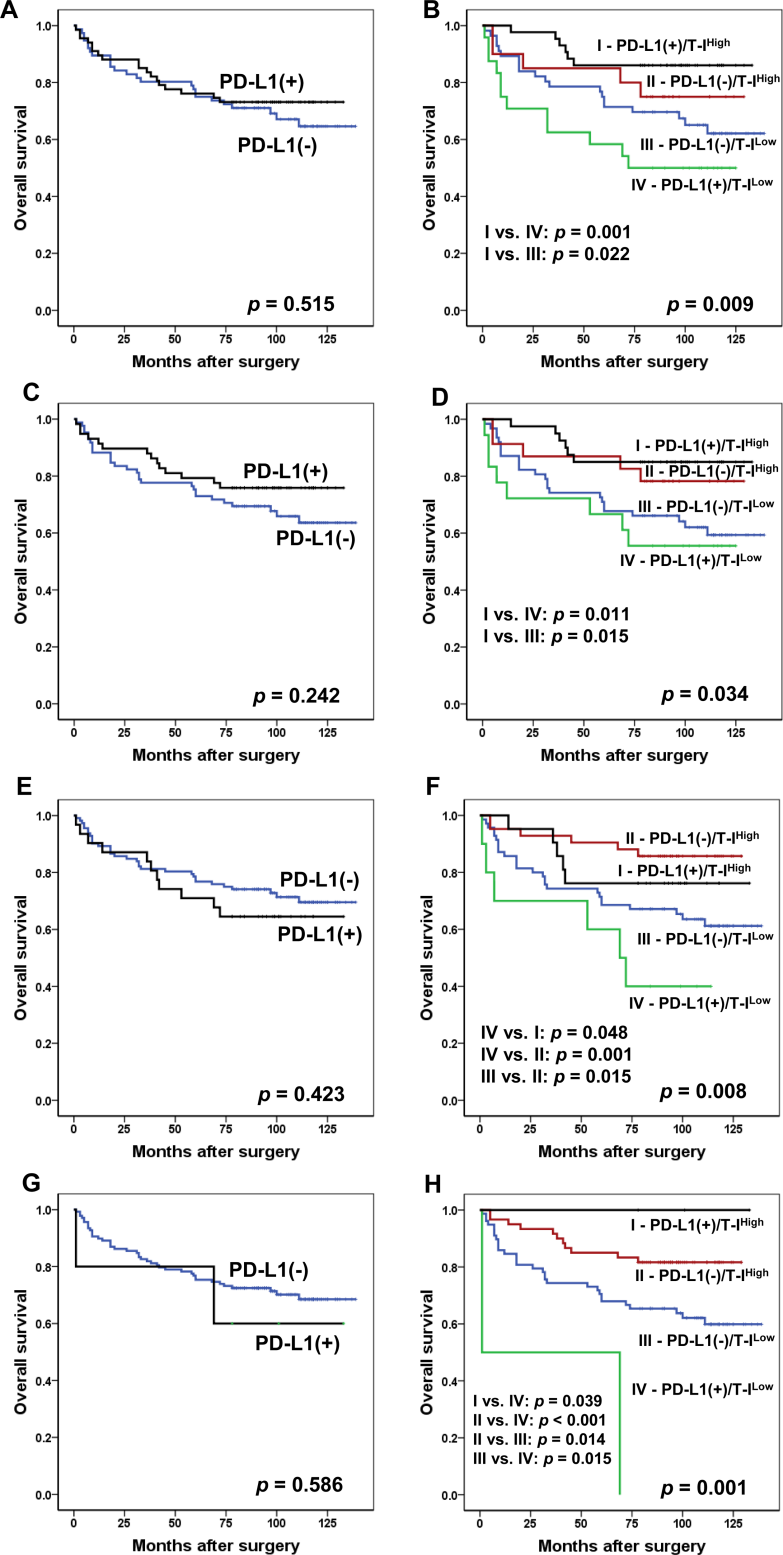
Kaplan-Meier survival analysis of the PD-L1 and PD-L1/T-I combination for 1% cut-off value (**A** and **B**), 5% cut-off value (**C** and **D**), 10% cut-off value (**E** and **F**) and 50% cut-off value (**G** and **H**). (A) Survival curves for OS according to PD-L1 expression status (No. of patients; PD-L1 (+), 67; PD-L1 (−), 76). (B) Survival curves for OS according to the PD-L1/T-I combination (No. of patients; PD-L1 (+)/T-I^High^, 43; PD-L1 (−)/T-I^High^, 20; PD-L1 (−)/T-I^Low^, 56; PD-L1 (+)/T-I^Low^, 24). (C) Survival curves for OS according to PD-L1 expression status (No. of patient; PD-L1 (+), 58; PD-L1 (−), 85). (D) Survival curves for OS according to the PD-L1/T-I combination (No. of patients; PD-L1 (+)/T-I^High^, 40; PD-L1 (−)/T-I^High^, 23; PD-L1 (−)/T-I^Low^, 62; PD-L1 (+)/T-I^Low^, 18). (E) Survival curves for OS according to PD-L1 expression status (No. of patients; PD-L1 (+), 31; PD-L1 (−), 112). (F) Survival curves for OS according to the PD-L1/T-I combination (No. of patients; PD-L1 (+)/T-I^High^, 21; PD-L1 (−)/T-I^High^, 42; PD-L1 (−)/T-I^Low^, 70; PD-L1 (+)/T-I^Low^, 10). (G) Survival curves for OS according to PD-L1 expression status (No. of patients; PD-L1 (+), 5; PD-L1 (−), 138). (H) Survival curves for OS according to the PD-L1/T-I combination (No. of patients; PD-L1 (+)/T-I^High^, 3; PD-L1 (−)/T-I^High^, 60; PD-L1 (−)/T-I^Low^, 78; PD-L1 (+)/T-I^Low^, 2). Abbreviations: T-PD-L1, PD-L1 expression in tumor cells; I-PD-L1, PD-L1 expression in immune cells; OS, overall survival; T-I, total immunoscore.

We then accounted for an integrated expression of T-PD-L1 and I-PD-L1 for the evaluation of comprehensive PD-L1 expression status in the tumor microenvironment. When the tumors were positive for either T-PD-L1 or I-PD-L1 at each cut-off value, they were classified into the “PD-L1 (+) group” while the remainders were classified into the “PD-L1 (−) group”. According to the Kaplan-Meier survival analysis, no significant association was found between PD-L1 expression and OS (*p* = 0.515 for 1% cut-off value; *p* = 0.242 for 5% cut-off value; *p* = 0.423 for 10% cut-off value; *p* = 0.586 for 50% cut-off value) (Figure 4A, 4C, 4E and 4G). To determine whether a differential prognostic effect of PD-L1 depended on the immunoscore, a combined analysis of the T-I and PD-L1 variables was performed. According to the Kaplan-Meier analysis, significant survival differences were observed among the four subgroups (*p* = 0.009 for 1% cut-off value; *p* = 0.034 for 5% cut-off value; *p* = 0.008 for 10% cut-off value; *p* = 0.001 for 50% cut-off value) (Figure 4B, 4D, 4F and 4H). The best OS was observed in PD-L1 (+)/T-I^High^ patients, whereas, PD-L1 (+)/T-I^Low^ patients exhibited the worst OS, except for the 10% cut-off value at which PD-L1 (+)/T-I^High^ group showed the second best OS. In particular, a distinct difference was noted in the OS between the patients with PD-L1 (+)/T-I^High^ and PD-L1 (+)/T-I^Low^ tumors at all cut-off value (*p* = 0.001 for 1% cut-off value; *p* = 0.011 for 5% cut-off value; *p* = 0.048 for 10% cut-off value; *p* = 0.039 for 50% cut-off value) (Figure 4B, 4D, 4F and 4H). A multivariate analysis with a Cox proportional hazard regression model that included lymphatic invasion, vascular invasion, perineural invasion, Ming classification, TNM stage, and the combination of PD-L1 expression and the T-I, which were significant factors in the univariate analysis (Supplementary Table 1), was performed. The combined status of PD-L1 expression and the T-I was an independent and significant prognostic factor for OS at each 1%, 5% and 10% cut-off value in patients with MSI-H GC (*p* = 0.030 for 1% cut-off value; *p* = 0.024 for 5% cut-off value; *p* = 0.019 for 10% cut-off value) (Table 3), and notably, the patients with PD-L1 (+)/T-I^High^ tumors showed a significantly better clinical outcome than patients with PD-L1 (+)/T-I^Low^ tumors (*p* = 0.008 for 1% cut-off value; *p* = 0.007 for 5% cut-off value; *p* = 0.042 for 10% cut-off value) (Table 3). However, at 50% cut-off value, there was no statistical significance (data not shown).

**Table 3:** Multivariate analysis of OS among patients with MSI-H GCs including combined PD-L1/T-I

Variable	Multivariate analysis (1% cut-off value)			Multivariate analysis (5% cut-off value)			Multivariate analysis (10% cut-off value)		
	*n*	HR (95% CI)	*p*-value	*n*	HR (95% CI)	p-value	*n*	HR (95% CI)	*p*-value
Ming									
Expanding	37	1 (Reference)	0.087	37	1 (Reference)	0.076	37	1 (Reference)	0.09
Infiltrative	106	2.528 (0.875–7.306)		106	2.609 (0.906–7.514)		106	2.503 (0.868–7.218)	
Lymphatic invasion									
Absent	50	1 (Reference)	0.423	50	1 (Reference)	0.337	50	1 (Reference)	0.476
Present	93	1.416 (0.605–3.316)		93	531 (0.642–3.648)		93	1.374 (0.574–3.290)	
Vascular invasion									
Absent	120	1 (Reference)	0.068	120	1 (Reference)	0.048	120	1 (Reference)	0.031
Present	23	1.977 (0.952–4.106)		23	2.122 (1.007–4.472)		23	2.262 (1.079–4.744)	
Perineural invasion									
Absent	89	1 (Reference)	0.547	89	1 (Reference)	0.724	89	1 (Reference)	0.669
Present	54	1.219 (0.640–2.320)		54	1.124 (0.587–2.154)		54	1.150 (0.606–2.181)	
AJCC stage									
I/II	85	1 (Reference)	< 0.001	85	1 (Reference)	< 0.001	85	1 (Reference)	< 0.001
III/IV	58	4.447 (2.046–9.665)		58	4.868 (2.247–10.543)		58	4.691 (2.178–10.104)	
Combined PD-L1/T-I^a^									
PD-L1(+)/T-I^High^	43	1 (Reference)	0.030	40	1 (Reference)	0.024	21	1 (Reference)	0.019
PD-L1(−)/T-I^High^	20	1.310 (0.380–4.520)	0.669	23	1.113 (0.323–3.841)	0.865	42	0.569 (0.168–1.923)	0.364
PD-L1(+)/T-I^Low^	24	3.911 (1.431–10.689)	0.008	18	4.486 (1.494–13.469)	0.007	10	3.577 (1.047–12.222)	0.042
PD-L1(−)/T-I^Low^	56	1.840 (0.704–4.807)	0.214	62	1.794 (0.703–4.576)	0.221	70	1.297 (0.492–3.422)	0.599

^a^Included only for patients with available TMA data.

Abbreviations: OS, overall survival; GC, gastric cancer; MSI-H, microsatellite instability-high; HR, hazard ratio; CI, confidence interval; AJCC, American Joint Committee on Cancer; T-I, total immunoscore; TMA, tissue microarray.

### Comparison of PD-L1 expression using two different monoclonal antibodies (E1L3N and 28-8)

To validate whether E1L3N was appropriate for evaluation of the endogenous PD-L1 expression levels of these tumors, we analyzed the concordance of two different PD-L1 antibodies (E1L3N and 28-8). The frequency of the positive and negative expression of PD-L1 (28-8) at each cut-off value is shown in Figure 2. The concordance of T-PD-L1 and I-PD-L1 at each cut-off value between the two antibodies was evaluated (Supplementary Table 3). For T-PD-L1, moderate to (kappa: 0.41 – 0.60) substantial (kappa: 0.61–0.80) level of concordance was observed [19]. The assessment using the 1% cut-off value showed the best concordance correlation coefficient (kappa value, 0.74; *p* < 0.001), followed by the 5% cut-off value (kappa value, 0.71; *p* < 0.001). For I-PD-L1, the level of concordance was similar to that of T-PD-L1, with the highest concordance level at the 1% cut-off value (kappa value, 0.72; *p* < 0.001). Representative paired images between these two antibodies are demonstrated in Supplementary Figure 2.

## DISCUSSION

Many recent studies have revealed that the presence of inflammatory cells within the tumor microenvironment is associated with an improved clinical outcome and increased response to chemotherapy and radiotherapy [20]. The correlation of a high density of TILs and favorable prognosis has been demonstrated in various tissues and tumor types, including colorectal [16], breast [21], prostatic [22], and esophageal cancers [23]. Among many T lymphocyte subpopulations, CD3, CD8, and CD45RO-positive T cells were most frequently assessed in studies analyzing the effect of TILs on patient prognosis [24]. For comprehensive estimation of prognostic immune parameters, the concept of “immune contexture” was applied, which is defined by the type, density, functional orientation, and location of the immune cells within distinct tumor regions [20, 25]. To improve the utility of immune cell infiltration estimation in a clinical setting, several researchers established the “immunoscore” that is based on enumeration of two lymphocyte populations, CD3+ and CD8+ TIL, in the TC and IF of CRCs [20]. In our study, we found that high immunoscore correlated with prolonged OS and was a good independent prognostic indicator in MSI-H GCs. These findings support the hypothesis that immunoscore is a useful and reproducible tool for predicting survival for patients with MSI-H GCs [17]. In addition, it can be inferred that relatively good prognosis for patients with MSI-H tumors is attributed to some extent to the increased numbers of TIL. This argument is supported by a study of Galon *et al*. which demonstrated that the difference in survival was not significant between patients with MSS/MSI-L CRC with high immune-related gene expression and patients with MSI-H CRC [11]. They insisted that, in predicting patients’ survival, immunoscore is a stronger parameter than MSI. In this study, infiltrating immune cells were evaluated separately in two distinct compartments (E and S) for more objective results. This decision was because the distribution of TIL differs between the two compartments and the ratio of the stroma and epithelium varies among the cores of TMAs [26, 27].

The correlation between PD-L1 expression and microsatellite instability has been demonstrated in several studies. Recently, a phase II clinical trial showed that anti-PD-1/PD-L1 therapy can be beneficial to patients who have advanced stage MSI-H colorectal cancer (CRC) [10]. The correlation between MSI-H cancers and high PD-L1 expression is logical given that MSI-H tumors have an increased number of tumor infiltrating immune cells, particularly Th1 and cytotoxic T cells, as well as a higher expression of immune checkpoint molecules [11]. This phenomenon is due to many immunogenic neo-antigens that are produced by frequent frameshift mutations in MSI-H tumors. However, not all MSI-GCs harbor dense infiltration of TILs in the tumor microenvironment, and not all express a high level of PD-L1. Instead, a wide spectrum of TIL density and PD-L1 expression may exist within MSI-H GCs. Because MSI-H GCs are a relatively homogenous group in terms of molecular carcinogenesis, they might be good sources through which to assess the prognostic value of PD-L1 expression and its relationship to the tumor microenvironment.

In our study, PD-L1 expression was observed in tumor cells (*n* = 33, 23.1% for 5% cut-off value) as well as in infiltrating immune cells (*n* = 43, 30.1% for 5% cut-off value), and a substantial number of tumors showed co-expression of PD-L1 in both tumor cells and immune cells (*n* = 18, 12.6% for 5% cut-off value). Although the biological meaning of these differential expression patterns is elusive, they are likely governed by combined innate (intrinsic) and adaptive cellular (extrinsic) factors within the tumor microenvironment [28]. The expression of PD-L1 has been reported to be regulated by intrinsic and extrinsic mechanisms in the tumor microenvironment [29]. The extrinsic induction is basically dependent on the pro-inflammatory cytokine interferon gamma (IFN-γ), which is secreted by CD8+ cytotoxic T cells; consequently, this induces the expression and transcription of PD-L1 on the surface of tumor cells and infiltrating immune cells [30]. In contrast, the intrinsic induction of PD-L1 on the surface of tumor cells is mediated by constitutive oncogenic and transcriptional pathways, such as the PI3K and mTOR pathways in non-small cell lung cancer and the EGFR-MAP kinase pathway in breast cancer [29]. In our study, a significant correlation was observed between T-PD-L1 expression and a high immunoscore. These findings likely reflect that PD-L1 expression in tumor cells is mainly controlled by an extrinsic (adaptive immune) mechanism rather than an intrinsic pathway in MSI-H GCs. In this regard, the results of the study by Derks *et al*. corresponded with our results in that MSI-H GCs exhibited expression of high IFN-γ response genes compared with genetically stable GCs according to a gene set enrichment analysis [30]. However, our results contrast with those of the study by Kim *et al*. [31]. They showed that PD-L1 expression in tumor cells was not associated with the densities of immune cells (CD3+, CD4+, CD8+, and PD-1+ cells) in 243 cases of GC. The discrepancy between the two studies can be attributed to the heterogeneity present in the study by Kim *et al*.'s group, as their study might have contained MSS/MSI-L GCs in excess of MSI-H GCs. The discrepancy also reflects a fundamental difference in the expression mechanisms of PD-L1 on tumor cells between MSS/MSI-L and MSI-H GCs. In addition, a more significant correlation was noted between I-PD-L1 expression and a high immunoscore. These findings seem plausible considering that CD3+ TILs, CD8+ TILs, and PD-L1-expressing immune cells are inherent components of the adaptive immune system, which are robustly activated in MSI-H GCs.

To date, there has been no standardized scoring system for evaluation of PD-L1 expression in any tumor type, including GCs. A 5% cut-off value was widely accepted because this level was presumed reproducible with no inherent variability in the assay process [32]. However, many studies and clinical trials [33, 34] applied a wide range of cut-off levels (1% to 50%). On that basis, PD-L1 expression was characterized in this study using four different cut-off values (1%, 5%, 10% and 50%), and was evaluated for its role as a prognostic biomarker at each cut-off value. Some results in regards to the prognostic value of PD-L1 expression are conflicting. Although most previous studies revealed a correlation between high PD-L1 expression and reduced survival rate in GCs [35, 36], several recent studies revealed that high PD-L1 expression had a positive impact on patient survival in GCs [13, 31, 33] and CRCs [37]. In our study, no significant survival difference was observed between the PD-L1 (+) and PD-L1 (−) groups. However, a combined survival analysis of PD-L1 expression and the immunoscore revealed four distinct subgroups with statistically significant differences in the OS, at all cut-off values. Notably, the PD-L1 (+)/T-I^Low^ group showed the worst prognosis, at 1%, 5% and 10% cut-off values, and the PD-L1 (+)/T-I^High^ group showed the best prognosis, at 1%, 5% except for 10% cut-off at which PD-L1 (−)/T-I^High^ group showed the best prognosis. Based on previous research, PD-L1 (+)/T-I^High^ refers to the group in which the adaptive immune system is primarily activated via the PD-l/PD-L1 signaling pathway, whereas the PD-L1 (+)/T-I^Low^ group is considered to be associated with intrinsic induction of PD-L1 by an oncogenic pathway regardless of IFN-γ expression [29]. The reason why the PD-L1 (+)/T-I^High^ subgroup has the most favorable prognosis, contrary to our expectations, might be because of a compensatory up-regulation of PD-L1 mediated by an ongoing overloaded antitumor immune response, rather than because of tumor immune evasion itself [38, 39]. This indicates that anti-tumor cytotoxic T cells and their counteractive molecules such as PD-L1 are simultaneously activated in highly immunogenic conditions in which TILs are abundant. In addition, the prognostic role of PD-L1 might be influenced by the tension state of complex immune contextures. However, according to our findings, it is noteworthy that intrinsic PD-L1 expression (i.e., PD-L1 (+)/T-I^Low^), is most harmful to patients with MSI-H GC. The mechanism by which PD-L1 expression through the intrinsic pathway is associated with the most aggressive behavior has not been clearly elucidated. In addition, the efficiency of anti-PD-1/PD-L1 therapy for patients with tumors driven by the intrinsic pathway is less clear compared to those induced by the adaptive pathway. Although constitutive expression of PD-L1 in tumors is considered as the mechanism that is less likely to be responsive to anti-PD-1/PD-L1 therapy despite their strong and diffuse PD-L1 expression pattern [40], limited numbers of studies in Hodgkin's lymphoma demonstrated a positive correlation between PD-L1 expression in tumors lacking PD-L1 positive T cells and their response to anti-PD-1/PD-L1 therapy [41]. Using larger-scale cohorts of GC patients, future studies should clarify the role of the combination of PD-L1 expression and the presence of TILs in the tumor microenvironment as a putative prognostic indicator and as a predictive biomarker for the application of anti-PD-1/PD-L1 therapy.

At this time, companion (pembrolizumab) or complementary diagnostics (nivolumab and atezolizumab) using different antibodies (i.e.,22C3, 28-8, SP142) coupled with correspondent assay platforms are FDA-approved for selection of patients who are treatable with anti-PD-1/PD-L1 antibodies [42]. Recent studies revealed that some commercial antibodies available as laboratory-developed tests (LDT), including E1L3N, have proven to be concordant with FDA-approved antibodies and have equivalent sensitivity [42–44]. Although a quantitative protein assay such as western blotting is the most reasonable method to validate sensitivity and specificity for antibody use in IHC to detect innate PD-L1 expression levels, it could not be carried out because matched fresh tissue samples were not available. As an alternative, PD-L1 expression was determined by IHC using two different monoclonal antibodies (E1L3N, 28-8). These two antibodies displayed an appreciable overlap at each cut-off value. However, there was a difference in the degree of concordance at each cut-off value. In particular, the 1% cut-off value showed the highest concordance in T-PD-L1 (kappa value = 0.74; *p* value < 0.001) and I-PD-L1 (kappa value = 0.72; *p* value < 0.001). These results were in line with some previous studies. Schats *et al*. demonstrated that the 1% cut-off value showed a higher concordance rate than the 5% cut-off value in comparative analysis between E1L3N and SP142 antibodies in malignant melanoma [45]. Sun *et al*., in their study, demonstrated that E1L3N and 28-8 were highly concordant with each other in triple negative breast cancer and the 1% cut-off value had a higher kappa value than the 5% cut-off value, although the highest kappa value was observed for the 50% cut-off value [44]. Based on these results, adopting a 1% cut-off value is more reliable and clinically applicable for screening non-responders to anti-PD-1/PD-L1 agents in conditions where standardized assay protocols and scoring systems are not available [45]. However, several researchers insist that using negative PD-L1 expression as the exclusion criteria for anti-PD-1/PD-L1 antibody therapy has limitations because some patients who are PD-L1-negative also show a response to anti-PD-l/PD-L1 agents in clinical trials for non-small cell lung cancers [46]. The intratumoral heterogeneity could be a crucial factor that brings the discrepancy between actual response to anti-PD-l/PD-L1 agents and the prediction through evaluating PD-L1 expression status by IHC. In this regard, our study has limitation. Three TMA cores of tumors might not be sufficient to overcome this issue. Further studies recruiting more cores or using whole section are required to evaluate the precise meaning of PD-L1 expression as a prognostic marker in MSI-H GCs.

The correlation between the high density of TILs and a favorable prognosis has been demonstrated in various tissues and tumor types, including colorectal [16], breast [21], and prostate [22] cancers. In addition to its role as a prognostic marker, the presence of TILs is also recognized as a predictive biomarker for anti-PD-1/PD-L1 therapy. Tumeh *et al*. reported that, in patients with stage III malignant melanoma, the most predictive marker of clinical response to PD-1 blockade was the density of CD8+ TILs in the TC and IF and was not PD-1 or PD-L1 expression itself [47]. Considering this, a larger number of patients with MSI-H GC might be potential candidates for anti-PD-1/PD-L1 therapy regardless of their PD-1 or PD-L1 expression status. For more efficient use of anti-PD-1/PD-L1 antibodies in GC patients, a standardized set of criteria is required regarding several factors such as the types of antibodies and staining methods to be used, and the cut-off level for positivity of PD-L1 expression in terms of both intensity and extent. Another factor to consider is the cell type that will be evaluated among tumor cells and infiltrating immune cells (in patients with metastatic urinary bladder cancer, PD-L1 expression in tumor infiltrating immune cells was proven to have predictive value for anti-PD-L1 antibody treatment [48]).

In conclusion, our study revealed that the PD-L1 expression is primarily associated with an adaptive immune resistance mechanism and that the immunoscore can be a relevant marker in the determination of the prognostic role of PD-L1 expression in MSI-H GCs.

## MATERIALS AND METHODS

### Patients and specimens

In all, 153 formalin-fixed paraffin-embedded GC tissues were collected from the pathology archives of Seoul National University Hospital, Seoul, Korea. All tumor samples were derived from the patients who underwent radical surgical resection with extended lymph node (LN) dissection at our institution between 2004 and 2009. At our institution, MSI status was routinely evaluated in resected GC specimens by the molecular pathology laboratory. Among 1706 patients, 153 (8.7%) cases of advanced GC with MSI-H were identified. Clinicopathological information, including age, sex, tumor site, tumor differentiation, tumor depth, LN metastasis (LNM), presence of lymphatic, vascular and perineural invasion, TNM stage, Lauren classification, Ming classification, date of surgery, date of last follow-up, and date of recurrence or death, were collected retrospectively from the electronic medical records. We evaluated the OS, which is defined as the date of GC surgery to the date of death or the last clinical follow-up before December 31, 2015. The average OS time, which was 2552 days, ranged from 35 to 4241 days. In this study, death occurred in 46 (30.1%) cases out of all patients with MSI-H advanced GC. Histologic grading and tumor staging were based on the seventh edition of the AJCC Staging Manual. This study was approved by the institutional review board of Seoul National University Hospital.

### DNA extraction and determination of MSI

The methods used for the MSI analysis have been previously described [26]. Briefly, tumor samples that were manually microdissected were collected into 1.5-ml microtubes containing 50 μl lysis buffer (100 mM Tris-HCl, 0.5% Tween-20, 1 mM EDTA and 20 μg/ml proteinase K). The samples were then incubated for 24 to 48 hours at 55°C until the tissue-containing lysis buffer became clear. The proteinase K was inactivated by incubation at 95°C for 10 min. Extracted genomic DNA was stored at −20°C until further use. MSI status was assessed at the following loci according to the National Institutes of Health guidelines: *BAT25, BAT26, D2S123, D5S346*, and *D17S250*. We defined tumors as MSI-H when two or more markers showed instability and tumors as MSI-L when one marker showed instability; finally, we defined tumors as MSS when none of the markers was unstable.

### GC tissue microarray and immunohistochemistry

**Figure 5:**
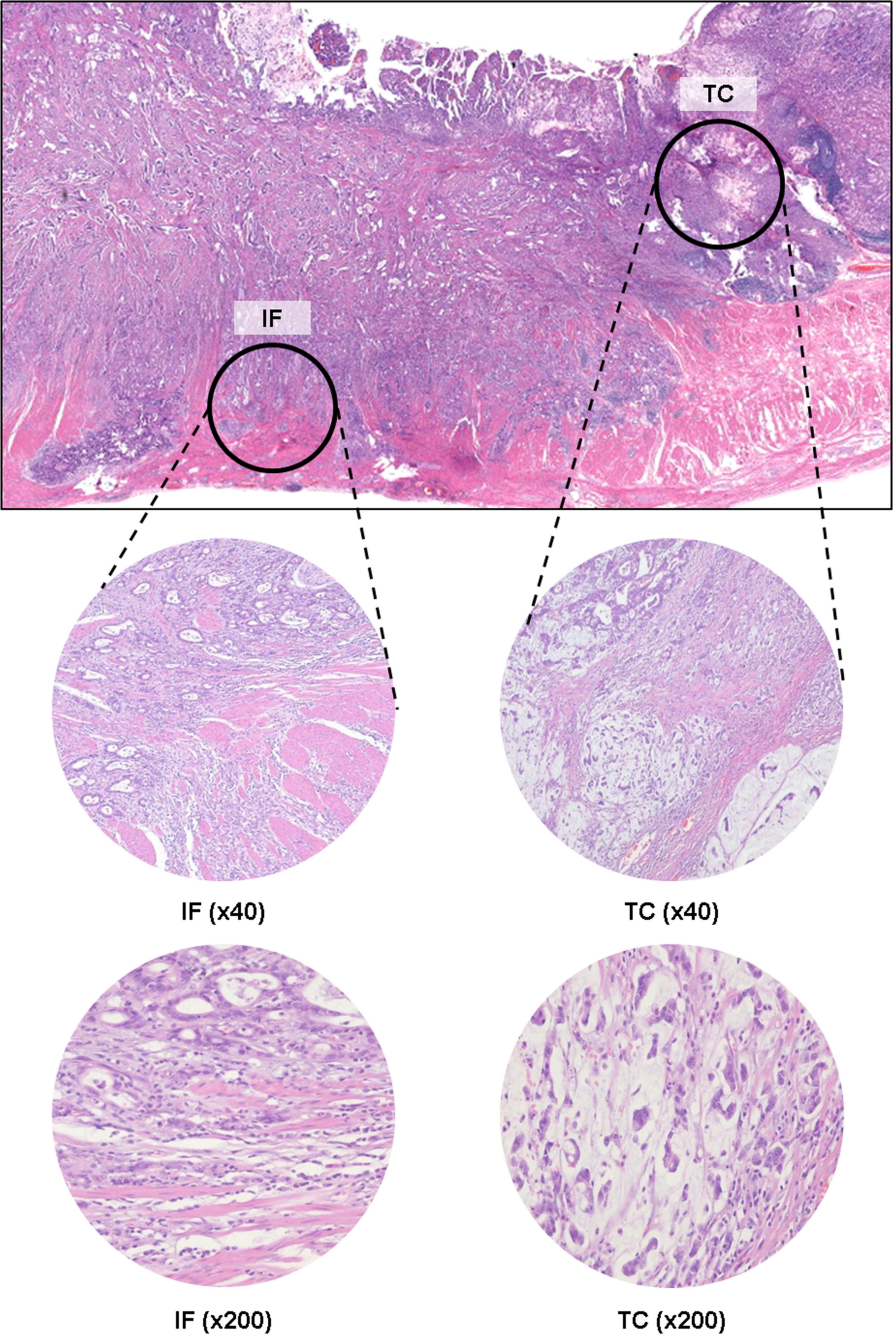
Representative image of the IF and TC in MSI-H advanced GCs H&E section of GCs (original magnification, 12.5×) (top) showing each regions of the tumor: IF and TC. Abbreviations: H&E, Hematoxylin and Eosin; IF, invasive front; TC, tumor center; MSI-H, microsatellite instability-high; GC, gastric cancer.

Tissue microarrays (TMAs) were constructed as previously described [26]. Tissue cores (2 mm in diameter) containing two representative tumor regions –tumor center (TC) and invasive front (IF) – were punched from individual donor tissue blocks and transferred to new recipient blocks using a trephine apparatus. One tissue core from the TC region and two cores from the IF regions were obtained from each case, and nine TMA blocks from 153 cases were constructed (Figure 5).

CD3, CD8, and two different PD-L1 clones (E1L3N and 28-8) were analyzed by immunohistochemical staining. In brief, the 4-μm thick, formalin-fixed, paraffin-embedded tissue sections were dewaxed in xylene and rehydrated using graded alcohol. Immunohistochemical staining for each marker was conducted using the BenchMark XT immunostainer (Ventana Medical Systems, Tucson, AZ, USA) under the following conditions: CD3 (rabbit polyclonal, 1:100; DAKO, Glostrup, Denmark), CD8 (SP16, 1:100; Neomarkers, Fremont, CA, USA), PD-L1 (E1L3N, 1:50; Cell Signaling Technology, Danvers, MA, USA), and PD-L1 (28-8, 1:50; Abcam, Cambridge, UK). The immunohistochemical staining protocol for each antibody is summarized in Supplementary Table 4.

### Determination of immunoscore

All immunostained TMA slides were scanned under high-power magnification (200×) using a scanner system (ScanScope XT; Aperio Technology, Vista, CA, USA). The Nuclear V9 algorithm of Image-Scope software (Aperio Technology) was used to evaluate the densities of CD3+ or CD8+ lymphocytes as numbers of CD3+ or CD8+ lymphocytes divided by the total area of selected foci (cells per square millimeter) (Supplementary Figure 1). One core of IF and TC regions were analyzed for determination of the CD3+ and CD8+ TIL densities. The density of CD3+ or CD8+ TILs was analyzed separately in the epithelial (E) and stromal (S) compartments within the TC and IF regions in the same core, which revealed TIL densities in four different areas (E-TC, S-TC, E-IF, and S-IF). Median, mean, and interquartile range (IQR) values of the density of each TIL type are listed in Table 4. The median values of CD3+ or CD8+ TIL densities were used as the cut-off to separate the low and high densities of TILs in each area. The immunoscores (I) of E and S compartments were assessed and designated as E-I and S-I, respectively. E-I and S-I were quantified by the number of high densities of CD3+ and CD8+ TILs in TC and IF regions within E and S compartments, respectively. Based on the combined CD3+ and CD8+ TIL densities in the two regions, the tumor was given a score ranging from 0 to 4 (E-I, E-TC + E-IF for CD3+ and CD8+ TILs; S-I, S-TC + S-IF for CD3+ and CD8+ TILs). The total immunoscore (T-I) was the sum of E-I and S-I and ranged from T-I0 to T-I8. For example, T-I8 refers to a tumor with high densities of CD3+ and CD8+ cells in the TC and IF regions within the E and S compartments, whereas T-I0 refers to a tumor with low densities of CD3+ and CD8+ cells. Supplementary Figure 1 shows how this scoring system was applied in this study. For further statistical analysis, the total immunoscore (T-I) was dichotomized as either T-I^Low^ (score of 0 to 4; *n* = 80) or T-I^High^ (score of 5 to 8; *n* = 63).

**Table 4:** Median, mean and IQR values of the density of CD3+ and CD8+ TIL in four different tumor areas

	Median^a^ (cells/mm^2^)	Mean^a^ (cells/mm^2^)	IQR^a^ (cells/mm^2^)	Range^a^ (cells/mm^2^)
E-TC CD3+ TIL	261	362	148 – 527	0 – 1343
S-TC CD3+ TIL	592	698	32 – 886	7 – 2310
E-IF CD3+ TIL	385	307	130 – 514	0 – 1350
S-IF CD3+ TIL	760	864	438 – 1299	0 – 3162
E-TC CD8+ TIL	229	305	99 – 449	0 – 1373
S-TC CD8+ TIL	384	405	154 – 548	0 – 1705
E-IF CD8+ TIL	315	387	207 – 552	0 – 1518
S-IF CD8+ TIL	462	569	223 – 781	0 – 2232

^a^Included only for patients with available TMA data.

Abbreviations: E, epithelial compartment; TC, tumor center; S, Stromal compartment; IF, invasive front; TIL, tumor infiltrating lymphocyte; IQR, interquartile range.

### Evaluation of PD-L1 expression

**Figure 6:**
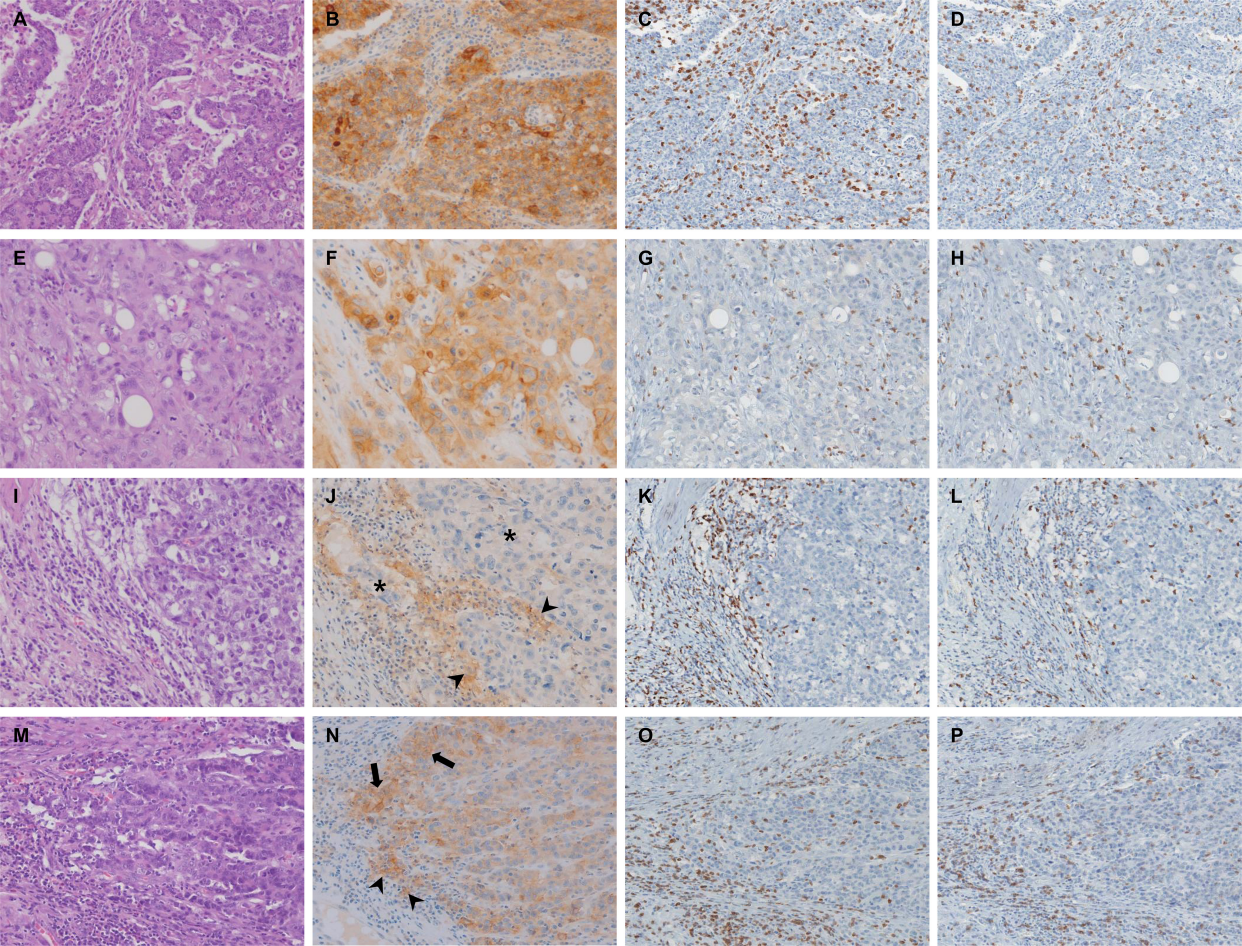
Immunohistochemical (IHC) staining results for PD-L1, CD3 and CD8 and matched H&E- stained samples (**A**–**D**) Representative histological features (A: H&E, 200×) and corresponding images of PD-L1 (B: IHC, 200x), CD3 (C: IHC, 200×) and CD8 (D: IHC, 200×). T-PD-L1 and I-PD-L1 expression was estimated to be positive in more than 50% and less than 1%, respectively. Immunoscore was T-I7 (E-I4 and S-I3). (**E**–**H**) Representative histological features (E: H&E, 200×) and corresponding images of PD-L1 (F: IHC, 200×), CD3 (G: IHC, 200×) and CD8 (H: IHC, 200×). T-PD-L1 and I-PD-L1 expression was estimated to be positive in more than 50% and less than 1%, respectively. Immunoscore was T-I1 (E-I0 and S-I1). (**I**–**L**) Representative histological features (I: H&E, 200×) and corresponding images of PD-L1 (J: IHC, 200×), CD3 (K: IHC, 200×) and CD8 (L: IHC, 200×). T-PD-L1 (asterisk) and I-PD-L1 (arrowhead) expression was estimated to be positive in less than 1% and about 25%, respectively. Immunoscore was T-I5 (E-I1 and S-I4). (**M**–**P**) Representative histological features (M: H&E, 200×) and corresponding images of PD-L1 (N: IHC, 200×), CD3 (O: IHC, 200×) and CD8 (P: IHC, 200×). T-PD-L1 (arrow) and I-PD-L1 (arrowhead) expression was estimated to be positive in about 10% and 5%, respectively. Immunoscore was T-I6 (E-I3 and S-I3). Abbreviations: T-PD-L1, PD-L1 in tumor cells; I-PD-L1, PD-L1 in immunce cells; H&E, Hematoxylin and Eosin; T-I, total immunoscore; E-I, immunoscore in epithelial compartment; S-I, immunoscore in stromal compartment; IHC, immunohistochemistry.

PD-L1 expression was assessed separately in tumor cells (T-PD-L1) and stromal immune cells (I-PD-L1) using the E1L3N clone. The intensity of membranous-to-cytoplasmic staining in tumor cells and stromal immune cells was initially scored on a scale of 0 to 3, as follows: negative (0), weak (1+), moderate (2+), and strong (3+). PD-L1 expression was determined to be positive when moderate (2+) and strong (3+) intensities were observed, and the extent of its expression was evaluated based on the proportion of positively stained tumor and immune cells. PD-L1 expression of tumor and immune cells was assessed by applying four cut-off values - 1%, 5%, 10%, and 50%. And it was defined as positive (PD-L1 (+)) when the number of cells positively stained for PD-L1 surpass that cut-off values. A highest score was recorded if three tissue cores from the same tumor exhibited different PD-L1 expression status. Representative images of PD-L1, CD3, and CD8 immunostaining with matched hematoxylin and eosin-stained samples are shown in Figure 6. For comparative analysis of the two PD-L1 expressing monoclonal antibodies, the expression status of PD-L1 by clone 28-8 was assessed in the same way as assessed for E1L3N.

### Statistical analysis

Categorical variables were compared using Pearson's chi-square test or Fisher's exact test (for cases with an *n* value < 10). A Kaplan-Meier log rank test was performed to compare the OS between the two subgroups. A multivariate survival Cox proportional hazards regression model was used to adjust variables that were statistically significant for the prognosis in the univariate analysis. The concordance between the two different PD-L1 antibodies (E1L3N and 28-8) was evaluated using Kappa-Cohen method. Statistical analysis was performed using IBM SPSS version 20.0 software (IBM Co., Armonk, NY, USA). All *P* values were two-sided, and *p* < 0.05 was considered statistically significant.

## SUPPLEMENTARY MATERIALS FIGURES AND TABLES




