# Supplementary material for: Combined prognostic effect of PD-L1 expression and immunoscore in microsatellite-unstable advanced gastric cancers
Source: Oncotarget. 2017 Jul 22;8(35):58887–902. doi: 10.18632/oncotarget.19439 (PMC5601701; doi:10.18632/oncotarget.19439)
Supplement: Supplementary file 1 [file oncotarget-08-58887-s001.pdf]

Combined prognostic effect of PD-L1 expression and immunoscore in microsatellite-unstable advanced gastric cancers

SUPPLEMENTARY MATERIALS

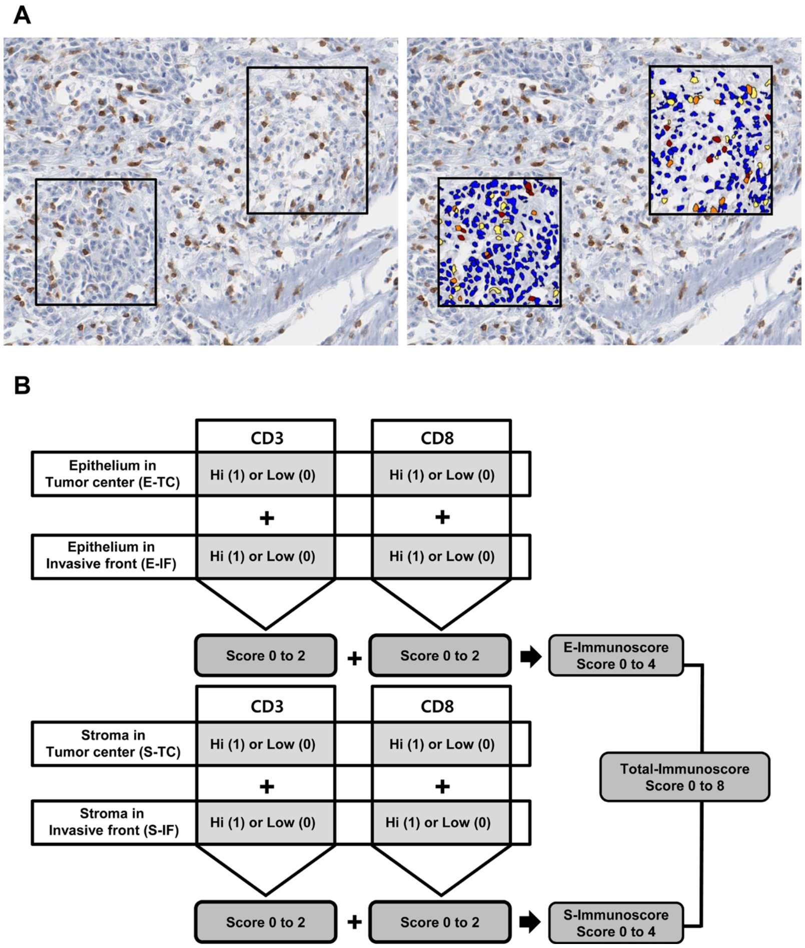

Supplementary Figure 1: Representative image of the counting the CD3+ TIL density and development of the immunoscore system. (**A**) By means of automatic image analysis system (ScanScope XT; Aperio), tumor density of positively stained cells was measured in E (left box in right figure) and S (right box in right figure) compartment. (**B**) The immunoscore ("I") of E and S compartments was separately assessed and designated as E-I and S-I, respectively. The E-I and S-I were quantified by the number of high densities of CD3+ and CD8+ TILs in E compartment of the TC and IF regions and S compartment of the TC and IF regions, respectively, ranging from 0 to 4. And then total immunoscore (T-I) which ranged from T-I0 to T-I8 was determined by sum scores of E-I and S-I. *Abbreviations*: TIL, tumor infiltrating lymphocyte; E-I, immunoscore in epithelial compartment; S-I, immunoscore in stromal compartment; T-I, total immunoscore; TC, tumor center; IF, invasive front.

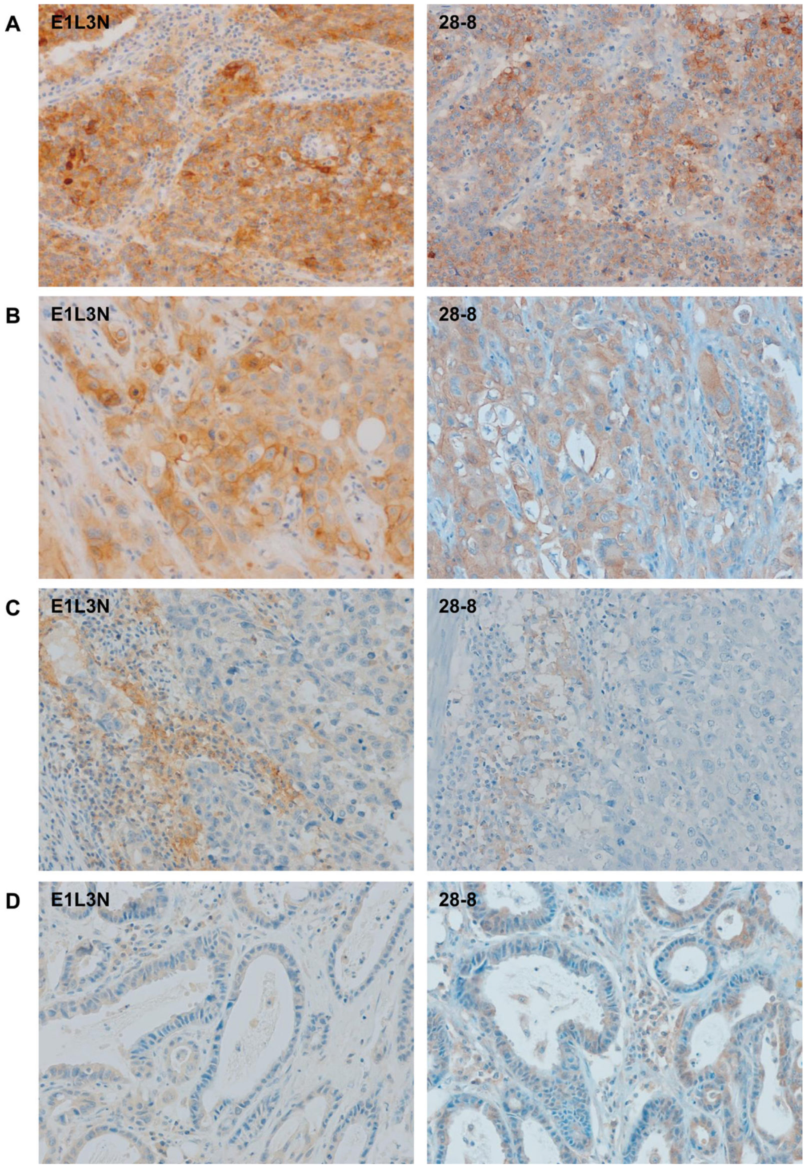

Supplementary Figure 2: Representative paired images of immunohistochemical staining of PD-L1 (E1L3N) and PD-L1 (28-8). (**A**) With use of E1L3N, T-PD-L1 and I-PD-L1 expression was seen in more than 50% of tumor cells and less than 1% of immune cells, respectively, whereas with use of 28-8, T-PD-L1 and I-PD-L1 expression was detected in about 15% of tumor cells and less than 1% of immune cells, respectively (200×). (**B**) T-PD-L1 and I-PD-L1 was expressed in more than 50% of tumor cells and less than 1% of immune cells, respectively for both E1L3N and 28-8 (200×). (**C**) T-PD-L1 and I-PD-L1 expression was estimated to be seen in less than 1% of tumor cells and about 25% of immune cells, respectively for E1L3N, and T-PD-L1 and I-PD-L1 was expressed in less than 1% of tumor cells and about 10% of immune cells, respectively for 28-8 (200×). (**D**) T-PD-L1 and I-PD-L1 expression was expressed in less than 1% of tumor cells and less than 1% of immune cells for E1L3N, whereas, T-PD-L1 and I-PD-L1 were expressed in about 20% of tumor cells and more than 50% of immune cells, respectively for 28-8 (200×). *Abbreviations*: T-PD-L1, PD-L1 expression in tumor cells; I-PD-L1, PD-L1 expression in immune cells.

Supplementary Table 1: Univariate analysis of OS among patients with MSI-H GCs. See Supplementary_Table_1

Supplementary Table 2: Summarized data representing *p*–value of Kaplan-Meier survival analysis for T-PD-L1 and I-PD-L1 at each cut-off value

	p – value of T-PD-L1	*p* – value of I-PD-L1
1% cut-off value	0.639	0.080
5% cut-off value	0.240	0.127
10% cut-off value	0.224	0.774
50% cut-off value	0.345	0.630

Abbreviations: T-PD-L1, PD-L1 expression in tumor cells; I-PD-L1, PD-L1 expression in immune cells.

Supplementary Table 3: Comparison of the PD-L1 expression between the two monoclonal antibodies (E1L3N and 28-8)

Cell component	T-PD-L1				I-PD-L1			
	1% cut-off	5% cut-off	10% cut-off	50% cut-off	1% cut-off	5% cut-off	10% cut-off	50% cut-off
kappa value (*p*-value)	0.74 (< 0.001)	0.71 (< 0.001)	0.55 (< 0.001)	0.66 (< 0.001)	0.72 (< 0.001)	0.68 (< 0.001)	0.67 (< 0.001)	0.49 (< 0.001)

Abbreviations: T-PD-L1, PD-L1 expression in tumor cells; I-PD-L1, PD-L1 expression in immune cells.

Supplementary Table 4: Immunohistochemical staining protocols of CD3, CD8, PD-L1 (E1L3N) and PD-L1 (28-8)

Antibody	CD3	CD8	PD-L1 (E1L3N)	PD-L1 (28-8)
Company	Dako	Neomarkers	Cell Signaling Technology	Abcam
Species	Rabbit	Rabbit	Rabbit	Rabbit
Clone	(−)	SP16	E1L3N	28-8
IHC platform	Ventana Benchmark XT	Ventana Benchmark XT	Ventana Benchmark XT	Ventana Benchmark XT
Antigen retrieval condition	CC1, 24 min (Ventana medical system, Tucson, AZ, USA)	CC1, 24 min (Ventana medical system, Tucson, AZ, USA)	CC1, 56 min (Ventana medical system, Tucson, AZ, USA)	CC1, 64 min (Ventana medical system, Tucson, AZ, USA)
Incubation time for primary antibody	16 min	16 min	32 min	56 min
Detection system	Optiview 3,3′-diaminobenzidine (DAB) detection kit (Ventana medical system, Tucson, AZ, USA)	Optiview 3,3′-diaminobenzidine (DAB) detection kit (Ventana medical system, Tucson, AZ, USA)	Optiview 3,3′-diaminobenzidine (DAB) detection kit (Ventana medical system, Tucson, AZ, USA)	Optiview 3,3′-diaminobenzidine (DAB) detection kit (Ventana medical system, Tucson, AZ, USA)
Dilution	1 : 100	1 : 100	1 : 50	1 : 50
Positive control	Tonsil	Tonsil	Tonsil, Placenta	Tonsil, Placenta

Abbreviations: IHC, immunohistochemistry.
