# Supplementary material for: Combined prognostic effect of PD-L1 expression and immunoscore in microsatellite-unstable advanced gastric cancers
Source: Oncotarget. 2017 Jul 22;8(35):58887–902. doi: 10.18632/oncotarget.19439 (PMC5601701; doi:10.18632/oncotarget.19439)
Supplement: Supplementary file 2 [file oncotarget-08-58887-s002.pdf]

Supplementary Table 1: Univariate analysis of OS among patients with MSI-H GCs

Variable	Univariate analysis		
	n	HR (95% CI)	*P*-value
Age			
≤ 60	48	1 (Reference)	0.296
> 60	105	0.723 (0.397-1.316)	
Tumor differentiation			
WD/MD	67	1 (Reference)	0.126
PD/Other	86	1.605 (0.875-2.945)	
Ming			
Expanding	40	1 (Reference)	0.004
Infiltrative	113	4.440 (1.589-12.408)	
Lymphatic invasion			
Absent	54	1 (Reference)	0.004
Present	99	3.084 (1.438-6.616)	
Vascular invasion			
Absent	129	1 (Reference)	0.019
Present	24	2.253 (1.143-4.441)	
Perineural invasion			
Absent	97	1 (Reference)	0.001
Present	56	2.754 (1.530-4.922)	
AJCC stage			
I/II	92	1 (Reference)	< 0.001
III/IV	61	7.975 (3.946-16.118)	
pT stage			
pT2/pT3	121	1 (Reference)	< 0.001
pT4	32	3.639 (2.013-6.577)	
pN stage			
pN0-N2	119	1 (Reference)	< 0.001
pN3	34	5.101 (2.847-9.139)	
T-PD-L1 expression (1% cut-off value)[a]			
Negative	98	1 (Reference)	0.640
Positive	45	0.853 (0.438-1.162)	
T-PD-L1 expression (5% cut-off value)[a]			
Negative	110	1 (Reference)	0.245
Positive	33	0.619 (0.275-1.391)	
T-PD-L1 expression (10% cut-off value)[a]			
Negative	126	1 (Reference)	0.230
Positive	17	1.644 (0.730-3.702)	
T-PD-L1 expression (50% cut-off value)[a]			
Negative	139	1 (Reference)	0.356
Positive	4	1.953 (0.472-8.077)	
I-PD-L1 expression (1% cut-off value)[a]			
Negative	98	1 (Reference)	0.086
Positive	45	0.525 (0.252-1.096)	
I-PD-L1 expression (5% cut-off value)[a]			

Negative	100	1 (Reference)	0.133
Positive	43	0.569 (0.273-1.187)	
I-PD-L1 expression (10% cut-off value)[a]			
Negative	125	1 (Reference)	0.873
Positive	18	0.873 (0.343-2.218)	
I-PD-L1 expression (50% cut-off value)[a]			
Negative	141	1 (Reference)	0.634
Positive	2	1.620 (0.222-11.799)	
Combined PD-L1 expression (1% cut-off value)[a]			
Negative	76	1 (Reference)	0.517
Positive	67	0.818 (0.446-1.501)	
Combined PD-L1 expression (5% cut-off value)[a]			
Negative	85	1 (Reference)	0.685
Positive	58	0.685 (0.362-1.297)	
Combined PD-L1 expression (10% cut-off value)[a]			
Negative	112	1 (Reference)	0.426
Positive	31	1.321 (0.665-2.624)	
Combined PD-L1 expression (50% cut-off value)[a]			
Negative	138	1 (Reference)	0.590
Positive	5	1.478 (0.357-6.112)	
Total-Immunoscore (T-I)[a]			
Low	80	1 (Reference)	0.007
High	63	0.389 (0.196-0.772)	
Combined PD-L1/T-I (1% cut-off value)[a]			
PD-L1(+)/T-I^{High}	43	1 (Reference)	0.017
PD-L1(-)/T-I^{High}	20	1.948 (0.594-6.383)	0.271
PD-L1(+)/T-I^{Low}	24	4.714 (1.767-12.571)	0.002
PD-L1(-)/T-I^{Low}	56	2.801 (1.124-6.978)	0.027
Combined PD-L1/T-I (5% cut-off value)[a]			
PD-L1(+)/T-I^{High}	40	1 (Reference)	0.048
PD-L1(-)/T-I^{High}	23	1.537 (0.469-5.036)	0.478
PD-L1(+)/T-I^{Low}	18	3.711 (1.287-10.701)	0.015
PD-L1(-)/T-I^{Low}	62	2.888 (1.180-7.071)	0.02
Combined PD-L1/T-I (10% cut-off value)[a]			
PD-L1(+)/T-I^{High}	21	1 (Reference)	0.016
PD-L1(-)/T-I^{High}	42	0.573 (0.175-1.880)	0.359
PD-L1(+)/T-I^{Low}	10	3.313 (1.011-10.863)	0.048
PD-L1(-)/T-I^{Low}	70	1.657 (0.636-4.321)	0.301

[a]Included only for patients with available TMA data.

Abbreviations: OS, overall survival; MSI-H, microsatellite instability-high; GCs, gastric cancers; HR, hazard ratio; CI, confidence interval; WD, well differentiated; MD, moderately differentiated; PD, poorly differentiated; T-PD-L1, PD-L1 expression in tumor cells; I-PD-L1, PD-L1 expression in immune cells; AJCC, American Joint Committee on Cancer; T-I, total immunoscore; TMA, tissue microarray.
